# miRNAs-19b, -29b-2* and -339-5p Show an Early and Sustained Up-Regulation in Ischemic Models of Stroke

**DOI:** 10.1371/journal.pone.0083717

**Published:** 2013-12-20

**Authors:** Dalbir K. Dhiraj, Elvina Chrysanthou, Giovanna R. Mallucci, Martin Bushell

**Affiliations:** MRC Toxicology Unit, Medical Research Council, Leicester, England, United Kingdom; University of British Columbia, Canada

## Abstract

Stroke, the loss of neurons after ischemic insult to the brain, is one of the leading causes of death and disability worldwide. Despite its prevalence and severity, current therapy is extremely limited, highlighting the importance of further understanding the molecular events underlying ischemia-induced neuronal cell death. An ischemic area can be subdivided into two separate pathophysiological regions: the rapidly dying necrotic core, and the potentially salvageable apoptotic penumbra. Understanding molecular events occurring in the apoptotic ischemic penumbra may give greater insight into mechanisms controlling this salvageable tissue. miRNAs are known to have key roles in the regulation of gene expression in numerous pathological conditions, including the modulation of distinct pathways in stroke. However, previous studies have profiled miRNAs in the whole ischemic infarct, and do not differentiate between miRNA regulation in the necrotic core versus the apoptotic penumbra. We asked if there were unique miRNAs that are differentially regulated following ischemic insults in the salvageable apoptotic penumbra. miRNA expression profiles were compared in the whole infarct from *in vivo* stroke models, using the three vessel occlusion approach, to an *in vitro* model of the ischemic penumbra, prior to apoptotic induction. Multiple miRNAs were found to be differentially regulated following ischemic insults in each system. However, miR-19b, miR-29b-2* and miR-339-5p were significantly up-regulated in both model systems. Further, we confirmed these results in a neuroblastoma cell line subjected to a penumbra-like ischemic insult that induced the apoptotic cell death pathway. The data show that miR-19b, miR-29b-2* and miR-339-5p are up-regulated following ischemic insults and may be regulating gene expression to control important cellular pathways in the salvageable ischemic penumbra. Further investigation of their role and mRNA target identification may lead to new insights into the molecular mechanisms taking place in the salvageable apoptotic penumbra.

## Introduction

Stroke is the third most common cause of death in the Western world and has a greater disability impact compared to other diseases [1]. The majority of strokes are ischemic, caused by a restriction of blood flow to a specific area of the brain [2]. Many drugs have been developed for the treatment of strokes, but have failed during clinical trials [3]; and thus current therapy is limited to the thrombolytic drug rt-PA (recombinant tissue-plasminogen activator). Often patients are not suitable candidates to receive this drug, since its use is restricted to the first 4.5 h after stroke [4]–[7]. This underscores the importance of further investigation into the molecular mechanisms underlying ischemia induced neuronal cell death for the development of novel therapeutics.

Ischemic stroke causes neuronal cell death that can be separated into two distinct regions, the core and penumbra [8]–[13]. The ischemic core experiences the greatest reduction in blood flow, receiving approximately 20% of its usual supply [8], [9], [12], [14]. Neurons within this region die within minutes to hours, mainly via necrotic mechanisms [10], [11], [13]. The area surrounding the core, the ischemic penumbra, is functionally silent but metabolically active and can account for up to half of the ischemic infarct at early stages [15], [16]. The penumbral neurons are able to repolarise following an ischemic event, however, this is carried out at the expense of energy consumption. Since the deficiency of oxygen and glucose delivery impairs the cells' ability to maintain ionic gradients, the neurons may depolarise. These events can cycle multiple times until the neurons are depleted of energy. Substantial evidence shows that cells in the penumbra will die over a period of hours to weeks mainly via an apoptotic pathway [8], [11], [12], [17]–[29].

Studies have identified alterations in post-translational modifications of BCL-2 family proteins following ischemic injury [30]–[35], indicating a role for the intrinsic pathway of apoptosis in the ischemic penumbra. Recent advances in developing better stroke therapies are now focussing on neuroprotective strategies that are targeted to the salvageable penumbra. This delayed apoptotic cell death has become the focus of many studies as it provides a larger window for effective therapy in comparison to the rapidly dying necrotic core.

MicroRNAs (miRNAs) have been identified to play an important role in the progression of neuronal death following cerebral ischemia [36]–[40]. MiRNAs are short non-coding RNA molecules that negatively regulate gene expression by base pairing with the 3′UTR of target mRNAs to inhibit both translation and decrease mRNA stability [41]–[49]. However, miRNA profiling data from ischemic models has often been generated at late time points, when cell death has occurred, and tissue samples used have included the rapidly dying necrotic core [36]–[40]. In order to understand the involvement of miRNAs in the apoptotic processes following stroke, this study focused on investigating the early post-transcriptional regulation of gene expression specifically in the ischemic penumbra.

The current study investigated changes in miRNA expression in rat cortical neurons exposed to an *in vitro* model of the ischemic penumbra (Oxygen Glucose Deprivation; OGD) before cell death was detected. In addition to this, miRNA microarrays were conducted on an *in vivo* model of transient cerebral ischemia at a time point where the salvageable apoptotic cell death of the penumbra is occurring. Our data suggest that miR-19b, miR-29b-2* and miR-339-5p are all up-regulated in response to ischemia, both *in vivo* and *in vitro*. These miRNAs were up-regulated before the detection of neuronal cell death (*in vitro*) and following establishment of the infarct (*in vivo*). Further, we also found that these miRNAs were up-regulated in neuroblastoma cells (N2As) following ischemic insults. This study highlights that miR-19b, -29b-2* and -339-5p are modulated in response to ischemic insults and may have a role in ischemia induced cell death.

## Materials and Methods

### Ethics Statement

All procedures were conducted in accordance with the UK Animals (Scientific Procedures) Act, 1986 (Home Office project licence 80/2323). The University of Leicester animal welfare committee approved all animal protocols. All surgery was performed under anaesthesia, efforts were taken to ameliorate suffering and mice were allowed free access to food and drink before and after surgery. Animals were humanely culled, by schedule 1 cervical dislocation, if they exceeded severity limits post surgery or at the end of the study. Wistar rats were fed and housed under standard conditions. Both adult and embryos were humanely culled using schedule 1 cervical dislocation.

### Primary cell culture

Cortical neurons were prepared from E17-18 embryos from Wistar rats. The cortex was extracted and the hippocampus was discarded, the tissue mechanically dissociated followed by enzymatic dissociation with 5% Trypsin + 0.05 mg/ml Dnase I at 37°C for 10 min. The tissue was centrifuged for 3 min at 1500 rpm and resuspended in Neurobasal medium supplemented with 10% heat inactivated FCS, 1x Glutamax, 50 Units/ml Penicillin and 50 µg/ml Streptomycin (Sigma). The neurons were seeded on to Poly-L-Lysine coated plates at a density of 8×10^5^ cells/ml. After 60 min, the plating medium was replaced with maintenance medium: Neurobasal medium supplement with 2% B27, 1x Glutamax, 50 Units/ml Penicillin and 50 µg/ml Streptomycin (Sigma). On 7 days *in vitro* (DIV), medium was supplemented with fresh maintenance medium and 10 µM cytosine arabinoside (Sigma) was added to prevent glial cell contamination.

### N2A cell culture

Mouse Neuroblastoma cell lines (N2As) were a kind gift from Miguel Martins (MRC Toxicology Unit, Leicester; originally purchased from ATCC [CCL-131]). They were maintained in DMEM + 10% FCS (Gibco). For OGD experiments, cells were seeded at 5×10^4^ cells/mL at least 48 h prior to OGD induction.

### Oxygen Glucose Deprivation

Cells were pre-treated with 10 µM DL-2-Amino-5-phosphonovaleric acid (APV) (Sigma) and 10 µM (+)MK-801 (Sigma) for 20 min. Neurons were subjected to OGD by replacing medium with glucose-free EBSS (116 mM NaCl, 5.37 mM KCl, 0.8 mM MgSO_4_, 1.17 mM NaH_2_PO_4_, 1.8 mM CaCl_2_, 26.19 mM NaHCO_3_) with the addition of 10 µM APV and 10 µM (+)MK-801. EBSS was gassed with 95% N_2_/5% CO_2_ for 30 minutes, in a water bath at 37°C. Neurobasal medium was removed from the neurons, replaced with deoxygenated glucose free EBSS and transferred to a pre-heated modular incubator chamber (Billups-Rothenberg, USA). The chamber was flushed with 95% N_2_/5% CO_2_ gas, sealed and placed into a 37°C incubator for 4 h. Anaerobic indicator sticks (BD) were used to confirm an anoxic environment within the chamber. Controls consisted of sister cultures in which medium was changed to EBSS + 10 mM Glucose, 10 µM APV and 10 µM (+)MK-801 and were placed in a normoxic incubator at 37°C. OGD was terminated by removing the EBSS and replacing it with fresh maintenance medium supplemented with 10 µM APV and 10 µM (+)MK-801. OGD experiments conducted with zVAD.fmk (MP Biomedicals) had a 20 min pre-treatment with the inhibitor, which was present in the OGD media and reperfusion media.

### 3 Vessel Occlusion (3VO) Stroke Surgery

The 3 vessel occlusion stroke model technique [50] was performed on C57BL/6J wild type mice (Charles River) at 8 to 14 weeks. Buprenorphine (Vetergesic) diluted in saline (0.1 mg/kg) was pre-operatively administered to the animals as an analgesic as well as for hydrating operated mice. Initial anaesthesia was induced with a mixture of 3 L/min isofluorane followed by maintenance anaesthesia at 1 to 2 L/min. O_2_ and N_2_O levels were kept constant at 1 L/min and 0.8 L/min respectively. Body temperature was maintained at around 36°C using a heating blanket. A ventral midline incision of the neck was made and the two common carotid arteries (CCA) were exposed followed by clamping of the left CCA using an aneurism clip. The left zygomatic arch was then removed to enable access to the skull and the middle cerebral artery. A 1 mm thick burr hole was opened 1 mm superior–rostral to the foramen ovale to allow access to the MCA followed by its permanent cauterisation using a bipolar coagulator (Aura, Kirwan Surgical Products). After the MCA occlusion, complete ischemia was induced for 30 minutes by clipping the right CCA. After the termination of ischemia both clips were removed allowing reperfusion for 24 h. Transient cerebral ischemia using this model results in unilateral cortical lesions affecting the left cortex. Sham operations performed included the whole procedure but without the dual common carotid artery occlusion and MCA cauterisation.

### Cell Death Assays

Cells were treated with 0.5 µM Sytox Orange nuclear stain (Invitrogen) (cell non-permeant, red fluorescence) and 5 µg/ml Hoechst 33342 (Invitrogen) (cell permeant, blue fluorescence) for 30 min at 37°C. The neurons were then washed once and placed in KREB/HEPES (KHB) buffer (130 mM NaCl, 5.4 mM KCl, 1 mM MgCl_2_, 1.8 mM CaCl_2_, 20 mM HEPES, 10 mM Glucose; pH 7.4). Imaging was conducted by epifluorescence microscopy.

For time-lapse imaging analysis, cells were treated with 1 µM Calcein AM (Molecular Probes) for 20 min, followed by a complete media change 12 h later. Cells were placed onto a heated stage microscope with CO_2_ control in a dark custom-built enclosure (Solvent Scientific) for 30 min before imaging was conducted. Staining was visualised using an Axiovert 200 M fluorescent microscope (Zeiss) and images were captured using a Hamamatsu Orca-ER camera using MetaMorph (Molecular Devices) software. Images were analysed to quantify the percentage of non-viable cells using Volocity 4.0 (Improvision).

### Western blotting

Protein analysis was conducted by Western blot as previously described [51]. Primary antibodies used: Cleaved Caspase 3, 1∶1000, Cell Signalling Technology (cat. 9664); Gapdh, 1∶10000, Santa Cruz (cat. Sc-365062); Cytochrome C, 1∶1000, BD Pharmingen (cat. 556432); PARP, 1∶1000, Cell Signalling Technology (cat. 9542); Erk (1/2), 1∶1000, Cell Signalling Technology (cat. 4695). Secondary antibodies used: Goat anti-mouse IgG (H+L) horse radish peroxidase conjugate and Goat anti-rabbit IgG (H+L) horse radish peroxidise conjugate, 1∶25,000 (Bio-Rad) for 1 h at RT, followed by final washes in TBST (4×10 min). Immuno-reactive proteins were detected by Enhanced Chemiluminescence Plus Western Blotting Detection System (Amersham). Immuno-reactivity was analysed using Image J software (NIH, http://rsbweb.nih.gov/ij/index.html).

To assess cytochrome C release from mitochondria into the cytoplasm, cells were collected using 0.05% Trypsin/0.5 nM EDTA, centrifuged at 200×g for 3 min, and then lysed for 5 min on ice (Lysis Buffer: 250 mM sucrose; 20 mM HEPES, 5 mM MgCl_2_; 10 mM KCl; 1 mM EDTA, 1 mM EGTA, 0.03% Digitonin and Protease inhibitor cocktail). The lysates were centrifuged at 13000 rpm at 4°C for 3 min and the supernatant (cytosolic fraction) was collected. A total of 5 µg of protein was loaded onto a 10% polyacrylamide gel, transferred onto nitrocellulose membrane and incubated with appropriate antibodies.

### MicroRNA microarrays

Total RNA was extracted using the miRNeasy mini Kit (Qiagen). The RNA 6000 Nano kit (Agilent) was used to assess RNA quality and integrity on an Agilent Bioanalyzer 2100. All samples were accepted on the basis that RNA integrity for each sample was >8.0.

Microarray slides were prepared in the laboratory of Dr. Tim Gant (MRC Toxicology Unit, Leicester) using the microRNA sequences from the miRBase v16.0 database (www.mirbase.org). Array slides were printed with probes, supplied by Exiqon (Denmark), complimentary to all mouse and rat microRNAs, including related viruses and controls. FlashTag 3DNA array detection kits (Genisphere, PA, USA) were used to label 1 µg of total RNA from control and ischemic samples (3 per group for *in vivo* and *in vitro* samples). Arrays were scanned using an Axon4200 scanner and data was compiled using GenePix Pro 6.0 (Molecular Devices, CA, USA). The microarray datasets can be viewed at Gene Expression Omnibus (www.ncbi.nlm.nih.gov/geo Accession Number: GSE51586).

### Taqman microRNA assays

Total RNA, including microRNAs, was isolated from primary neuronal cell cultures using miRNeasy Mini Kits (Qiagen). Reverse transcription was performed using the Taqman miRNA Reverse transcription kit (Applied Biosystems). Equal amounts of RNA (100 ng) were reverse transcribed, using 100 mM dNTPs, 50 U reverse transcriptase, 0.4 U RNase Inhibitor, and specific miRNA reverse transcriptase primers, with the following protocol: 16°C for 30 min, 42°C for 30 min, followed by 85°C for 5 min. PCR was then conducted on the reverse transcription (RT) products using the Taqman miRNA assay kit (Applied Biosystems). Each PCR contained 1.33 µL of the RT product, 10 µL of Taqman 2x Universal PCR Master Mix, 1 µL 20x Taqman microRNAs Assay Reagent in a total volume of 20 µL. The relative expression levels of miRNAs were normalised to endogenous U6 snRNA expression for each sample. Each RT product underwent PCR in triplicate. Validations using this method were conducted on the same RNA processed on the microarrays, with additional *in vitro* samples.

### Electron microscopy

Electron Microscopy was kindly performed by David Dinsdale (MRC Toxicology Unit, Leicester). In brief, cells were fixed in 2% glutaraldehyde in 0.1 M sodium cacodylate buffer (pH 7.4) at 4°C overnight and postfixed with 1% osmium tetroxide/1% potassium ferrocyanide for 1 h at room temperature. After fixation, cells were stained en bloc with 5% aqueous uranyl acetate overnight at room temperature, dehydrated, and embedded in Taab epoxy resin (Taab Laboratories Equipment Ltd., Aldermaston, UK). Ultrathin sections were stained with lead citrate and recorded using a Megaview 3 digital camera and iTEM software (Olympus Soft Imaging Solutions GmbH, Münster, Germany) in a Jeol 100-CXII electron microscope (Jeol UK Ltd., Welwyn Garden City, UK).

### Flow Cytometry

Cells were trypsinised and collected, including those floating in media. Total pooled cells (approximately 10^5^ in total) were centrifuged for 5 min at 2000 rpm at RT. Cells were washed in PBS and resuspended in 0.5 mL of annexin V binding buffer (10 HEPES, 150 mM NaCl, 5 mM KCl, 1 mM MgCl_2_, 1.8 mM CaCl_2_, pH 7.4). For each treatment group cells were left unstained or stained with Annexin V for 10 min and 0.2 µM Propidium Iodide (PI) for 2 min at RT. Cell samples, 10,000 events per sample, were analysed on a Becton Dickinson Canto II (BD Biosciences, USA) fitted with a 488 nm blue and 633 nm red lasers. Annexin-FITC emission was detected using a 530/30 nm filter and PI emission with a 585/42 nm filter. Negative controls and single Annexin V or PI stained cells were used to adjust voltages and compensation.

### Statistical analysis

Data is presented as a mean ± SEM, unless otherwise stated. Statistical significance was tested using an unpaired t-test (Graphpad Prism, Grapdhpad Software, USA). Statistical significance was accepted at p<0.05. * denotes p<0.05, ** denotes p<0.01 and *** denotes p<0.001.

## Results

### OGD induces a time-dependent increase in apoptotic neuronal cell death

Our study aimed to focus on the apoptotic component of ischemia-induced neuronal injury. Previous studies have reported that OGD is able to induce both necrosis and apoptosis [52], [53]. Necrotic cell death occurs due to the excessive release of glutamate, which binds to NMDA-receptors leading to an excitatory response. Therefore, to minimise the level of necrotic cell death within these cultures, neurons were pre-treated with NMDA-receptor antagonists APV and (+)MK-801 [54]–[56].

Before proceeding, we assessed the degree of cell death in our primary cortical neurons in normal culture conditions to be 24±2% - a level of basal death that is frequently observed in 10–11 DIV primary neuronal cultures [57]–[60]. To establish the appropriate duration of an OGD insult required to induce neuronal cell death, cortical neurons were exposed to control EBSS containing oxygen and glucose (EBSS+Oxygen+Glucose) or EBSS depleted of oxygen and glucose (OGD) for 2, 4 or 6 h. To terminate the OGD, the cells were placed in their normal maintenance medium for 24 h, at which point cell death analysis was conducted using Sytox/Hoechst.

**Figure 1:**
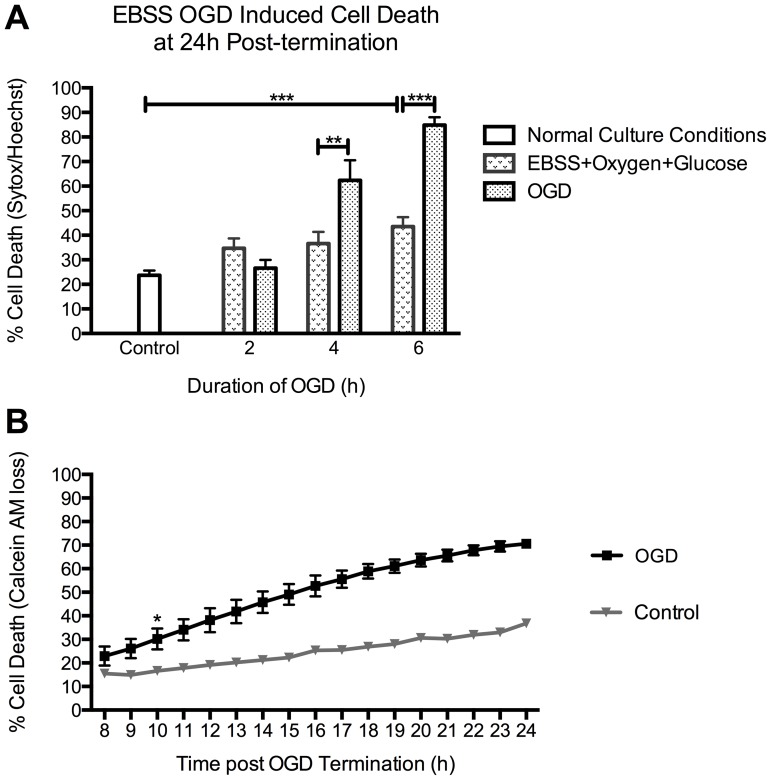
OGD-induced cell death in rat cortical neurons. A. OGD caused an increase in neuronal cell death levels, analysed by sytox/hoechst imaging, which correlated with increased durations of OGD. Basal levels of cell death within our cultures, under normal culture conditions, were determined as 23.7±2% (n = 3). Neurons were placed in EBSS with or without oxygen and glucose. Cells with EBSS plus oxygen and glucose (EBSS+Oxygen+Glucose) only demonstrated a significant increase in levels of death, in comparison to controls, following 6 h of exposure. 2 h of OGD did not increase cell death levels above those in EBSS+Oxygen+Glucose controls. However, 4 and 6 h of OGD increased cell death levels significantly to 62.4±8.2% (p = 0.026, n = 5) and 84.9±3.2% (p<0.0001, n = 5) respectively, compared to EBSS+Oxygen+Glucose controls. B. Cell death induced by 4 h of OGD becomes apparent between 8 and 24 h post-OGD termination. Analysis of the time course of OGD-induced cell death using Calcein AM time-lapse assay. Time-lapse imaging of Calcein AM stained cells indicated that OGD-induced cell death became apparent at 8 h and reached maximum levels at 24 h post-OGD termination. Levels of cell death became significantly higher than controls at 10 h post- OGD termination, indicated by *, where cell death levels were 18.7±2.4% and 39.7±6.4% for control and OGD, respectively (p = 0.045, n = 3). Neuronal cell death levels, following OGD reach 75±2% at 24 h post-OGD termination. Data represent mean ± SEM.

EBSS containing oxygen and glucose significantly increased cell death levels following a 6 h exposure, when compared to normal culture conditions (Figure 1A). The results indicated 27±3% (n = 3), 62±8% (p = 0.02, n = 5) and 85±3% (p<0.0001, n = 5) death in cultures exposed to 2, 4, and 6 h of OGD, respectively (Figure 1A). As we wished to investigate early events associated with ischemic injury, the minimum duration of OGD required to induce cell death was selected; hence subsequent experiments were conducted with the use of a 4 h OGD insult. For all subsequent experiments we have referred to the EBSS containing oxygen and glucose cohort as Controls.

Previous results had shown that cell death became apparent 8 h after termination of the OGD insult (data not shown). To investigate the time for cell death to manifest itself following an OGD insult, rat cortical neurons were imaged from 8 h to 24 h post-OGD termination (Figure 1B). Cortical neurons were incubated with 1 µM calcein AM for 20 min prior to OGD to visualise the proportion of viable cells at hourly intervals. The data demonstrated that neuronal cell death became significantly higher than controls at 10 h post-OGD termination; where controls had 19±2% and OGD-treated neurons had 40±6% cell death (p = 0.04, n = 3). OGD induced cell death levels rose at a steady rate reaching 75±2% at 24 h post-termination.

**Figure 2:**
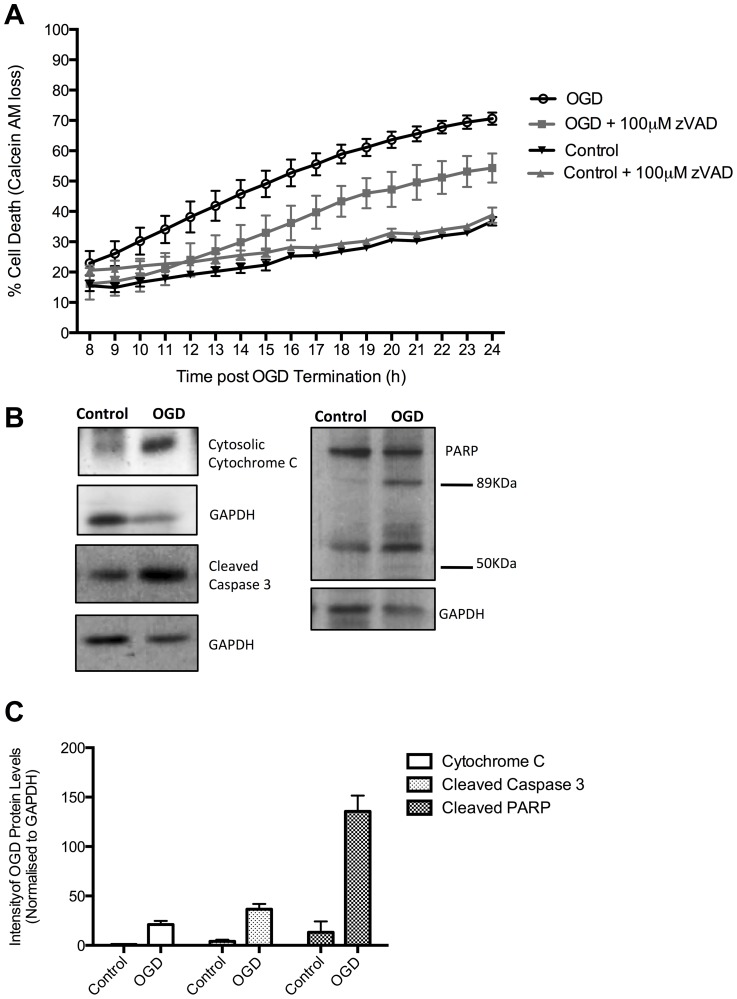
OGD induced cell death demonstrates characteristics of apoptotic cell death. A. The pan-caspase inhibitor, zVAD.fmk at a concentration of 100 µM, was able to significantly reduce levels of OGD-induced cell death from 70.6±2%, in naïve cells, to 54.3±5% in zVAD.fmk treated cells (p = 0.0459, n = 3) at 24 h post-OGD termination, thus indicating cell death is caspase dependent. B. Western blot analysis shows that OGD induces cytochrome C release into the cytosolic fraction of cell lysates. OGD also induces activation of Caspase 3, as indicated by the presence of the cleaved 17 kDa fragment. OGD-treated rat cortical neurons display PARP that has been cleaved to form the classic 89 kDa apoptosis fragment. The 50 kDa fragment of PARP that is found during necrosis is not detected. C. Quantification of the Western blots demonstrates an increase of cytochrome C intensity of 0.9±0.3, in controls, to 21±3.7 following OGD (p = 0.0056, n = 3). Quantification of Caspase 3 signal intensity shows a significant increase in OGD samples reaching 36.6±5.4 (p = 0.0046, n = 3). Quantification revealed that levels of the 89 kDa PARP fragment changed from 13.3±11 in control cells, to 135.6±16 in OGD treated rat cortical neurons (p = 0.0032, n = 3).Data represent Mean ± SEM.

To determine the pathway of OGD- induced neuronal cell death we next sought to assess whether death was dependent on caspases. OGD was conducted with the addition of 100 µM zVAD.fmk, a pan-caspase inhibitor (Figure 2A). We observed a general decrease in levels of cell death following treatment with zVAD.fmk. Cell death at 24 h post-OGD termination was significantly reduced to 54±5%, compared to naïve OGD treated cells (p = 0.04, n = 3). The results suggested that OGD-induced neuronal cell death is partially established by the activation of caspases.

Additionally, Western blot analysis conducted in order to identify characteristic hallmarks of apoptotic cell death revealed an increase in cytoplasmic Cytochrome C following OGD exposure (Figure 2B and 2C). Similarly, we observed increased levels of caspase 3 and PARP cleavage, classical apoptotic markers. The PARP Western blot analysis also revealed an absence of 50 kDa cleavage products that are indicative to necrosis [61] (See also Figure S1). These data indicate that OGD-induced neuronal cell death is predominantly apoptotic and therefore mimicks neuronal cell death in the ischemic penumbra.

### Ischemia induces the differential regulation of microRNAs

Previous studies investigating the role of miRNAs following ischemic insults have focused on time points where cell death had already occurred, without differentiation of the miRNA expression pattern between the apoptotic penumbra and necrotic core, where the latter infarct area cannot be completely rescued [36]–[40]. As it is important to establish the differences in cellular response within these two regions, this study aimed to investigate the regulation of miRNAs in the potentially salvageable mainly apoptotic ischemic penumbra. We assessed the miRNA profile of neurons at 8 h post-OGD termination, before the onset of neuronal cell death (Figure 1B) and where no nuclear chromatin condensation, a hallmark of apoptotic cell death, was yet observed (Figure S2). In addition to this we also analysed the regulation of miRNAs in RNA extracted from the ipsilateral cortex of mice exposed to 3-vessel occlusion (3VO) following 24 h of reperfusion, an *in vivo* model of stroke [48]. This model represented a more physiological system where the infarct had already been established (Figure S3). MiRNA regulation within the necrotic area would presumably have ceased, since the cells would have died early on and would have been removed by microglial mediated phagocytosis [80]–[81]; however, the penumbral area should still be mostly viable at this time point.

**Figure 3:**
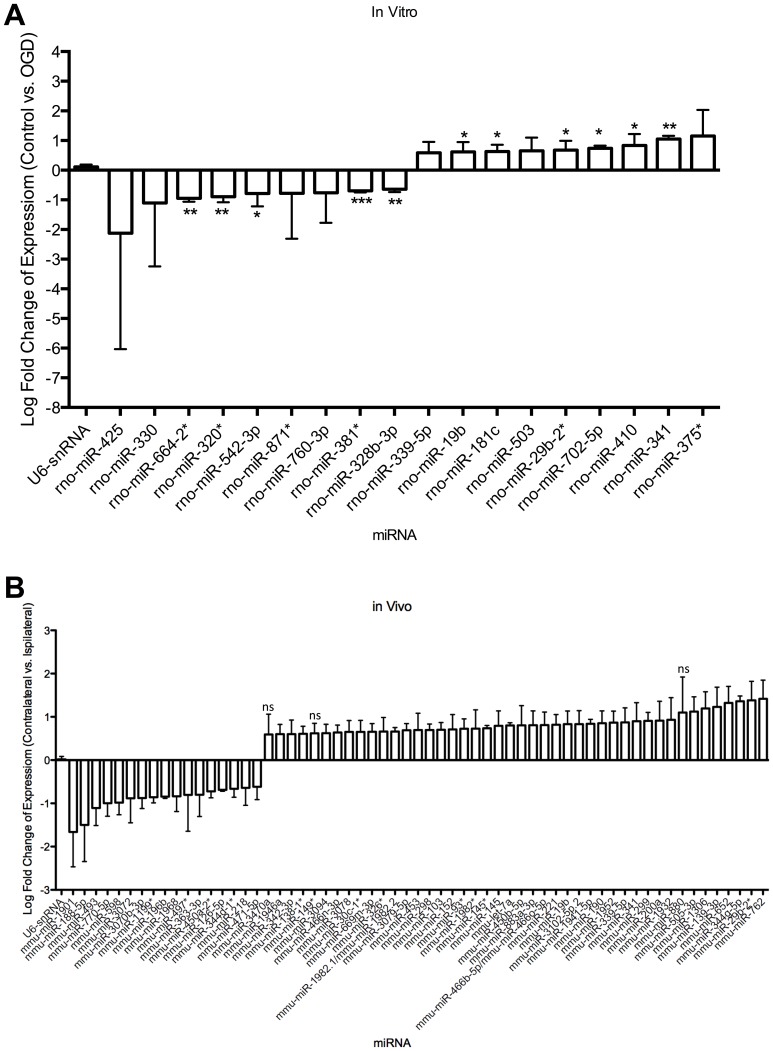
Ischemia induced the differential regulation of miRNAs both *in* vitro and *in* vivo. MiRNAs were selected on the basis that their expression changed by at least 1.5-fold (linear fold change) and are present in all biological repeats (n = 3) and each internal microarray technical repeat (4), *in vitro* (A) and *in vivo* (B). Data represents mean ± SD. Ns  =  non-significant. Significance accepted at p<0.05.

The results of the microarray analysis indicated that a number miRNAs were differentially regulated following ischemic insults both *in vitro* and *in vivo* (Figure 3). MiRNAs considered for further study were required to demonstrate an absolute fold change greater than 1.5 compared to controls, consistently in all biological and technical repeats. We identified 18 differentially regulated miRNAs in *in vitro* samples, of which 5 were significantly down regulated (miR-664-2*, miR-320, miR-542-3p, miR-381* and miR-328b-3p) and 6 miRNAs that were significantly up-regulated (miR-19b, miR-181c, miR-29b-2*, miR-702-5p, miR-410 and miR-341). The *in vivo* data suggested that 3VO caused a significant down-regulation in 17 miRNAs and a significant up-regulation of 41 miRNAs in the ipsilateral (positive for infarct) cortex compared to the contralateral (Control) cortex.

**Figure 4:**
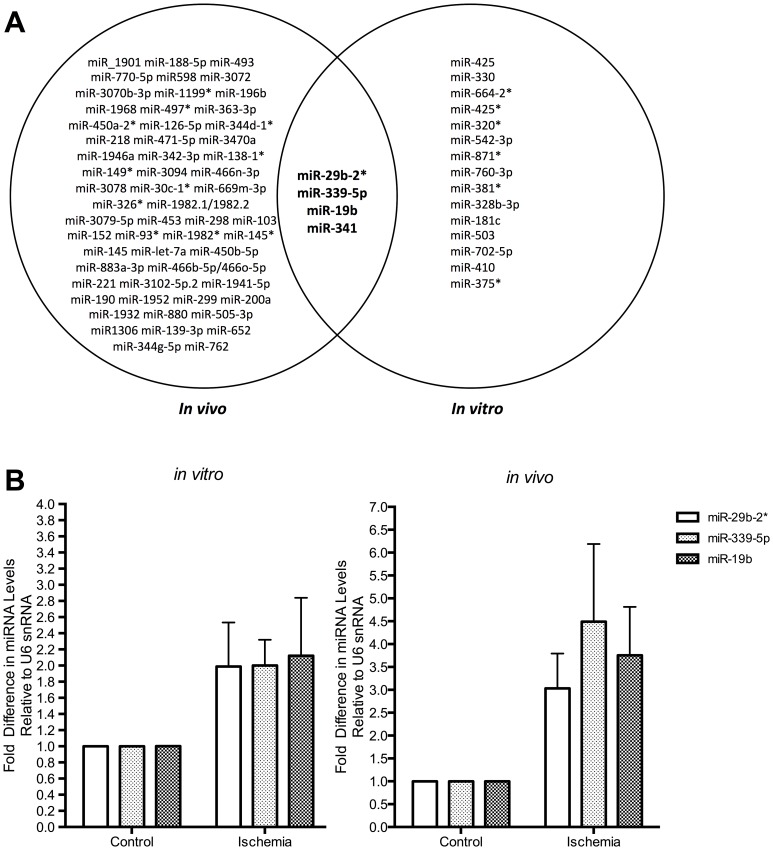
miR-29b-2*, miR-339-5p and miR-19b are up-regulated in response to *in vivo* and *in vitro* ischemia. A. Comparing both *in vivo* and *in vitro* data identified 4 miRNAs common to both models, which are differentially regulated in response to ischemia. B. Taqman Assay q-PCR confirms the up-regulation of miR-29b-2*, -339-5p and -19b following ischemic insults both *in vivo* (n = 3) and *in vitro* (n = 4).

The comparison of differentially expressed miRNAs in the *in vitro* model of the apoptotic penumbra and the *in vivo* brain revealed four miRNAs to be dysregulated in both systems: miR-19b, miR-339-5p, miR-29b-2* and miR-341 (Figure 4A). The validation of these changes by RT-qPCR (Figure 4B) demonstrated that miR-19b was up-regulated by 3.8±1 (p = 0.01, n = 3) and 2±0.7 (p = 0.02, n = 4)-fold in ischemic samples compared to control samples *in vivo* and *in vitro*, respectively. miR-339-5p was found to increase its expression by 4.5±1.7-fold *in vivo* (p = 0.02, n = 3) in the ipsilateral cortex when compared to the contralateral side. This miRNA was up-regulated by 2±0.3 fold (p = 0.02, n = 4) in cortical neurons exposed to OGD, compared to control neurons. Furthermore, miR-29b-2* was confirmed to be increased in ischemic *in vivo* and *in vitro* samples by 3±0.8 (p = 0.01, n = 3) and 2±0.6 (p = 0.02, n = 4)-fold, respectively. Validation of miR-341 was not performed due to lack of seed sequence conservation between mice and rats. Taken together, these data confirmed that miR-19b, miR-339-5p and miR-29b-2* are up-regulated before neuronal death is detected, *in vitro*, and following 24 h of reperfusion, *in vivo*.

We also generated a model of the apoptotic penumbral neurons in N2A cell lines, as this model system would allow the modulation of miRNAs to deduce their mechanism of action in future studies. The cells were exposed to 4 h of OGD and cell death was observed significantly higher than controls at 2 h post-OGD termination, reaching levels of 47.6±0.8% (p = 0.0006, n = 3) (Figure S4). We also confirmed by Western blot and electron microscopy that OGD-induced N2A cell death was apoptotic (Figure S5).

**Figure 5:**
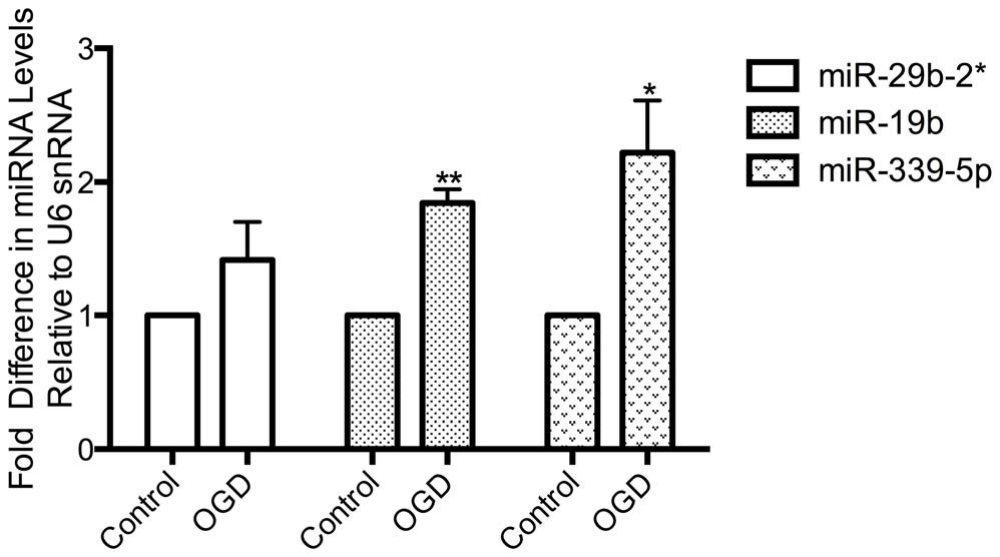
OGD in N2As induces the up-regulation of miR-29b-2*, miR-339-5p and miR-19b. Taqman q-PCR analysis of RNA collected 2 h post-OGD termination was analysed for the expression of selected miRNA candidates. The data shows that the miRNAs are up-regulated: by 1.42±0.28, 1.84±0.10 (P = 0.0013) and 2.22±0.39-fold (P = 0.035), for miR-29b-2*, miR-19b and miR-339-5p respectively (n = 3). Data represent mean ± SEM.

RT-qPCR analysis of miRNA levels in the N2A cells exposed to OGD showed that miR-19b, miR-339-5p and miR-29b-2* were all up-regulated in OGD treated cells in comparison to controls, by 1.8±0.1 (p = 0.01, n = 3), 2.2±0.4 (p = 0.04, n = 3) and 1.4±0.3 (n = 3)-fold, respectively (Figure 5). Taken together, our data strongly suggests that these miRNAs are differentially regulated directly in response to ischemia and not by inherent factors within each model system. We show that miR-19b, miR-339-5p and miR-29b-2* are all up-regulated in response to ischemic insults in 3 model systems: *in vivo* transient ischemia, *in vitro* OGD and N2A OGD. These miRNAs are up-regulated before neuronal death is detected (*in vitro*) and when cell death has been established (*in vivo* and N2A). These candidates represent targets for future research to elucidate their mechanism of action, mRNA targets and role following neuronal ischemia.

## Discussion

The present study investigated some aspects of the early regulatory events associated with the induction of neuronal cell death in the ischemic penumbra. Since current stroke therapy is restricted to a narrow therapeutic window, this study focused on ischemic death in the penumbra that can occur several hours or weeks after the initial insult, to potentially widen the window for intervention. Using OGD, we developed an *in vitro* model of the apoptotic penumbra in rat cortical neurons. We showed that OGD was able to induce apoptotic cell death; confirmed by the detection of characteristic apoptotic hallmarks such as cytochrome C release, caspase 3 activation and PARP cleavage (Figures 1 and 2). Further, we found that the inhibition of caspases, using the pan-caspase inhibitor zVAD.fmk, was able to partially reduce levels of cell death. These results support previous studies that have identified protection against ischemia-induced cell death following caspase inhibition in *in vivo* models [62]–[64]. The lack of complete protection against OGD induced death observed in this study is most likely due to caspase-independent cell death occurring in the presence of zVAD.fmk [65]–[71]. Together, our *in vitro* OGD treatments represent a model of apoptotic cell death observed in the ischemic penumbra.

Our study aimed to investigate early regulatory events associated with ischemia-induced cell death in the salvageable apoptotic penumbra, with a focus on regulation of gene expression carried out by miRNAs. The physiological role of miRNAs in the brain has been well documented [72]–[76]; furthermore there is compelling evidence that these non-coding regulatory elements are implicated in the onset and progression of neurodegenerative conditions [36], [37], [38], [77], [78], [79].

Previous studies have provided confirmation for the role of miRNAs following cerebral ischemia [36]–[40]. The current study has focused on the regulation of miRNAs before neuronal cell death has occurred when miRNA-based regulation would still be operational. We conducted microarray analysis of miRNAs in the established OGD model, before neuronal death was detected (Figure 1B, 3 and S2), thus focusing on an early penumbric event. In order to detect miRNAs that are differentially regulated in a more physiologically relevant model, we also assessed miRNAs in RNA extracted from mice exposed to the 3VO model of cerebral ischemia, at a time point where cell death had already been established (Figure 3 and S3) and the cells of the necrotic core are likely to have been rapidly removed by microglia [80], [81], leaving cells undergoing apoptosis. Together, this allowed the identification of miRNAs that are regulated in response to ischemic insult in the apoptotic penumbra.

Our data shows that 4 miRNAs were up-regulated in response to ischemia both *in vivo* and *in vitro* (Figure 4). We were also able to validate the changes observed for miR-19b, miR-339-5p and miR-29b-2*, which have previously been shown to be differentially regulated following ischemia in animal models and stroke patients [36]–[38]. Importantly, we observe these changes in both mouse and rat derived model systems (Figure 3 and 4) suggesting an evolutionarily conserved molecular function. Interestingly, at least one of these miRNAs, miR-19b, has been identified as up-regulated in human stroke patients [36]. These data also suggest that although the three models used in this study involve different neuronal populations, our identification of common miRNAs show that similar pathways are being affected by the ischemic insult. Together, the results imply that these miRNAs could have a role in the neuronal response to ischemic insults, before apoptotic cell death occurs in the ischemic infarct. In addition to this, we also confirmed that these 3 miRNAs are up-regulated in response to OGD in N2A cells (Figure 5), which confirms that the change is indeed a response to ischemia rather than a result of other inherent factors within each model system. Furthermore, confirmation of the up-regulation in N2A cells means that these miRNAs could be modulated in a simple cell line system to deduce their mechanism of action.

Prediction of miRNA targets can be challenging, as in some instances mRNA targets are not seed-matched [82]. Using prediction algorithm software, such as Targetscan (http://www.targetscan.org/), one can predict some of the mRNA targets of miRNAs. miR-19b, -339-5p or-29b-2* were not predicted to directly target any regulators of apoptosis, such as the BCL-2 family, though this does not exclude such a possibility. Nevertheless, they could potentially regulate cell death by acting on mRNAs upstream of the activation of the apoptotic cascade.

The data highlight the importance of identifying the biological function, targets of these miRNAs and pathways that they impact to obtain a better understanding of whether they are driving neuronal cell death following ischemia, or whether they are acting as a pro-survival signal that eventually fails. These insights will allow the assessment of their utility as potential therapeutic targets in efforts to salvage the ischemic penumbra.

## Supporting Information

Figure S1 **OGD without the addition of APV and (+)MK-801 induces immediate and caspase-independent cell death.** A. Cell death was analysed immediately after the termination of OGD (0 h post-OGD Termination) using Sytox/Hoechst staining. Neurons pre-treated with 10 µM APV and (+)MK-801 did not show any increase in cell death levels in comparison to controls. However, without the addition of these NMDA-receptor antagonists, cell death levels were significantly increased to 74.4±1.62% (p<0.001, n = 3). B. 100 µM zVAD.fmk does not protect against OGD-induced cell death in the absence of APV and (+)MK-801 when analysed at 24 h post-OGD termination (n = 3).
(TIF)

Figure S2 **OGD-induced condensed chromatin is not present at 8 h post termination.** Hoechst staining of neuronal nuclei was used to identify condensed chromatin. Neurons exposed to OGD showed no increase in condensed chromatin, in comparison to controls, at 8 h post-OGD termination. However, condensed chromatin was visible at 24 h post-termination. Representative images of n = 3. Scale bar  = 10 µm.
(TIF)

Figure S3 **3VO surgery induces neuronal cell death in the mouse ipsilateral cortex.** 2, 3, 5- Triphenyltetrazolium Chloride (TTC) staining, a metabolic cell indicator of mitochondrial activity, was conducted on representative coronal brain sections from 3-VO operated and sham operated mice. Mouse brains were sliced into 1 mm thick sections and were stained with 2% TTC in saline, at room temperature for 30 minutes in the dark. The sections were then fixed in 10% formalin (Sigma) and stored in the dark at 4°C. The results indicate that cortical lesions (non-coloured, indicated by arrows) are developed following 3-VO when assessed following 24 h of reperfusion. Stained areas represent healthy, non-infarcted tissue.
(TIF)

Figure S4 **EBSS induced OGD is toxic to N2A cells.** A. N2As were exposed to a 4 h OGD insult using EBSS. Cell death was analysed at 0, 2 and 4 h post-OGD termination by examining Annexin V and PI incorporation using flow cytometry. The data indicates that total levels of cell death become significant at 2 h post-OGD termination (47.63±0.79%), in comparison to the 0 h time point (21.85±2.47%) (p = 0.0006, n = 3). These cell death levels are maintained when analysed at 4 h post-OGD termination (50.55±3.14%, n = 3). In comparison, control cells are unaffected by equivalent treatments, in the presence of oxygen and glucose. (n = 3). Data represent mean ± SEM.
(TIF)

Figure S5 **OGD in N2As induces cell death that displays characteristic hallmarks of apoptosis.** A. N2As exposed to 4 h of OGD were examined for PARP cleavage and generation of the cleaved active caspase 3 fragment by Western analysis. The representative blots illustrate that OGD has induced a substantial increase in the cleaved PARP product of 89 kDa, characteristic of apoptosis, in comparison to the 0 h time point. Furthermore, this cleavage product is still present when examined at 4 h post OGD termination. Western analysis for the 17 kDa active cleavage product of caspase 3 also showed a large increase in its abundance at 2 h post-OGD termination, in comparison with the 0 h sample. The 17 kDA cleavage product declined in levels by 4 h post-OGD termination, when compared to the 2 h sample. The diagram represents a representative image from n = 3. B. N2As exposed to OGD show morphological characteristics of apoptosis. Electron microscopy was utilised to identify morphological hallmarks of apoptosis at 2 and 4 h post-OGD termination. Control images depict representative images of healthy cells. A small number of cells appear to be lysed (open arrow), illustrating basal death levels in the culture. At 2 h post-OGD termination cells with budding membranes (closed arrow) and condensed chromatin (dashed arrow) can be detected. Budding membranes and condensed chromatin are also detected at 4 h post OGD termination. (n = 1) Scale bar  = 5 µm.
(TIF)
