# Supplementary figures and images for: miRNAs-19b, -29b-2* and -339-5p Show an Early and Sustained Up-Regulation in Ischemic Models of Stroke
Source: PLoS One. 2013 Dec 20;8(12):e83717. doi: 10.1371/journal.pone.0083717 (PMC3869799; doi:10.1371/journal.pone.0083717)

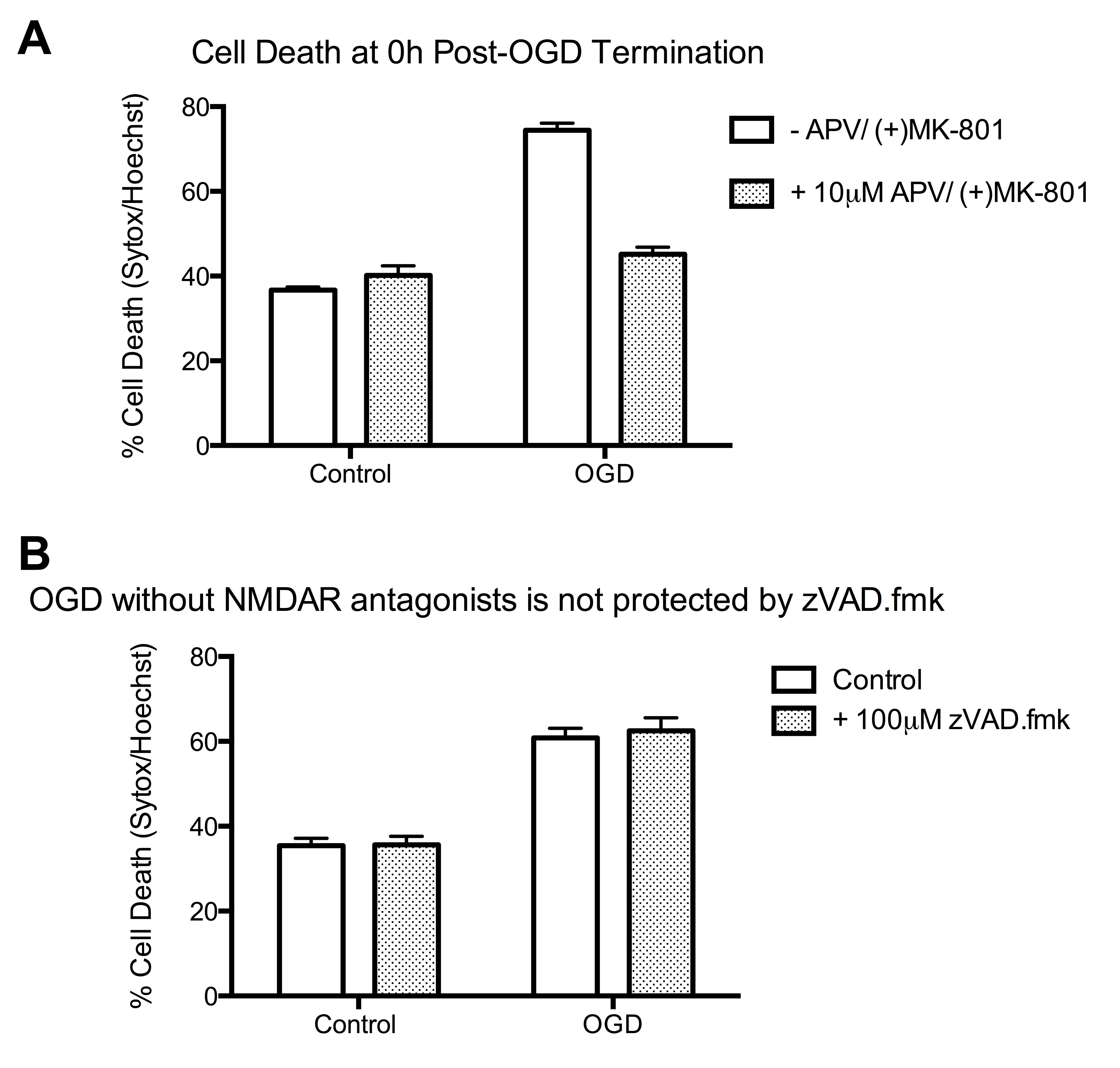

Supplement: Figure S1 — OGD without the addition of APV and (+)MK-801 induces immediate and caspase-independent cell death. A. Cell death was analysed immediately after the termination of OGD (0 h post-OGD Termination) using Sytox/Hoechst staining. Neurons pre-treated with 10 µM APV and (+)MK-801 did not show any increase in cell death levels in comparison to controls. However, without the addition of these NMDA-receptor antagonists, cell death levels were significantly increased to 74.4±1.62% (p<0.001, n = 3). B. 100 µM zVAD.fmk does not protect against OGD-induced cell death in the absence of APV and (+)MK-801 when analysed at 24 h post-OGD termination (n = 3). (TIF) [file pone.0083717.s001.tif]

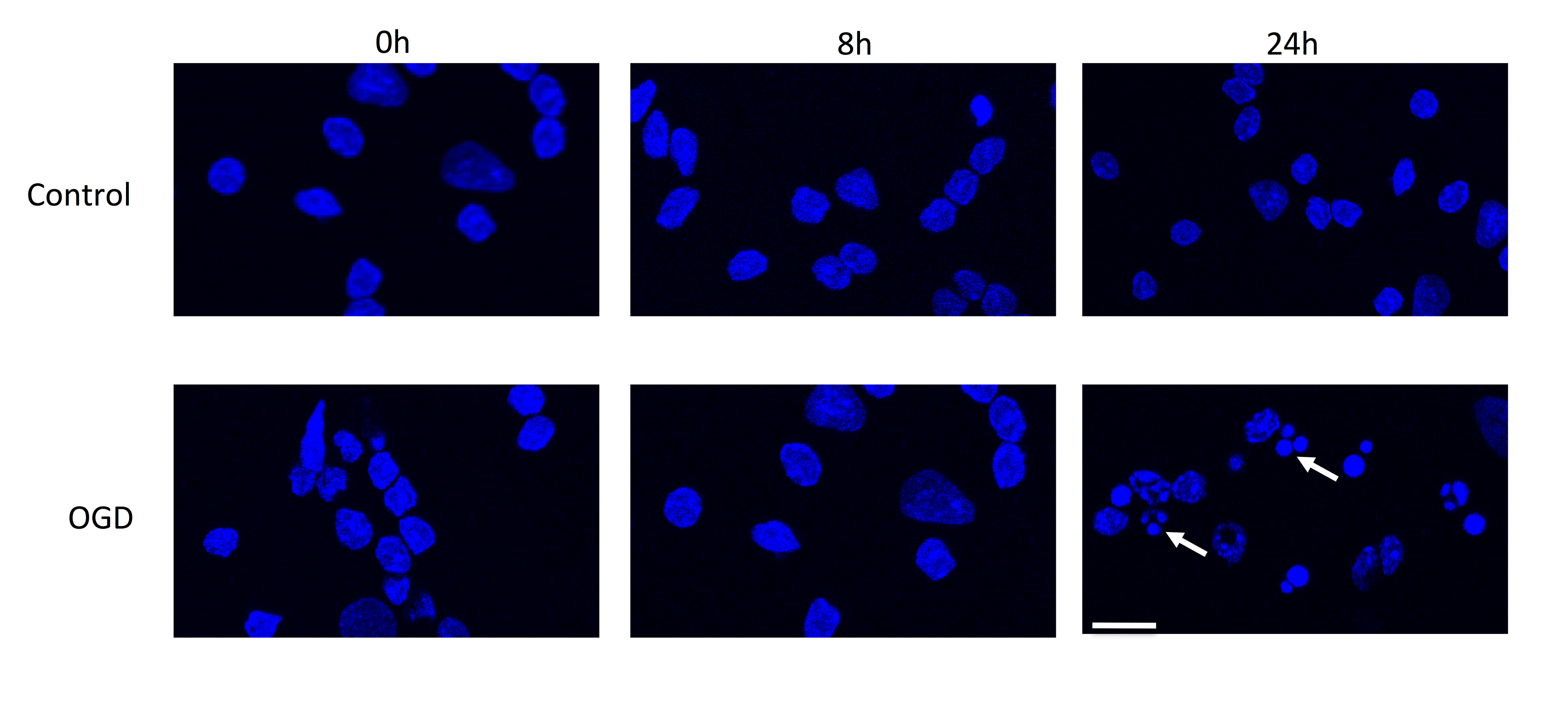

Supplement: Figure S2 — OGD-induced condensed chromatin is not present at 8 h post termination. Hoechst staining of neuronal nuclei was used to identify condensed chromatin. Neurons exposed to OGD showed no increase in condensed chromatin, in comparison to controls, at 8 h post-OGD termination. However, condensed chromatin was visible at 24 h post-termination. Representative images of n = 3. Scale bar = 10 µm. (TIF) [file pone.0083717.s002.tif]

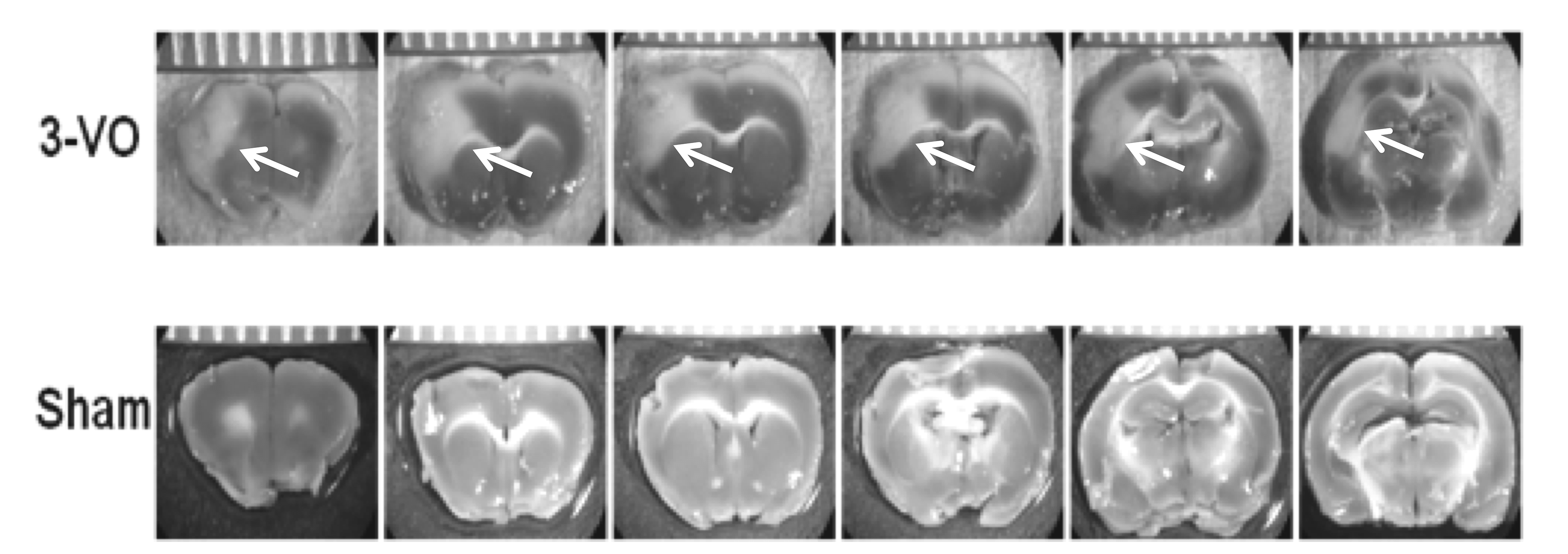

Supplement: Figure S3 — 3VO surgery induces neuronal cell death in the mouse ipsilateral cortex. 2, 3, 5- Triphenyltetrazolium Chloride (TTC) staining, a metabolic cell indicator of mitochondrial activity, was conducted on representative coronal brain sections from 3-VO operated and sham operated mice. Mouse brains were sliced into 1 mm thick sections and were stained with 2% TTC in saline, at room temperature for 30 minutes in the dark. The sections were then fixed in 10% formalin (Sigma) and stored in the dark at 4°C. The results indicate that cortical lesions (non-coloured, indicated by arrows) are developed following 3-VO when assessed following 24 h of reperfusion. Stained areas represent healthy, non-infarcted tissue. (TIF) [file pone.0083717.s003.tif]

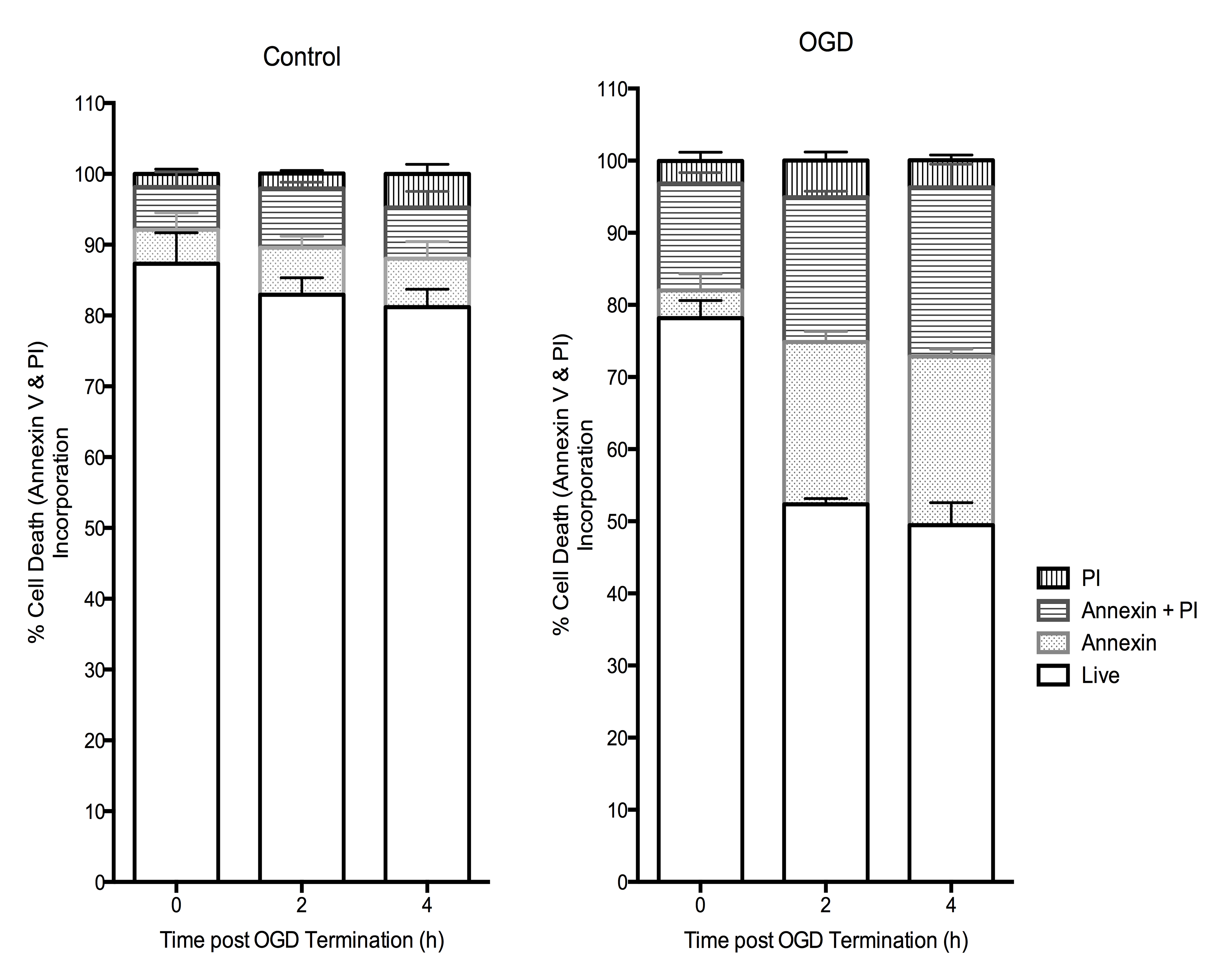

Supplement: Figure S4 — EBSS induced OGD is toxic to N2A cells. A. N2As were exposed to a 4 h OGD insult using EBSS. Cell death was analysed at 0, 2 and 4 h post-OGD termination by examining Annexin V and PI incorporation using flow cytometry. The data indicates that total levels of cell death become significant at 2 h post-OGD termination (47.63±0.79%), in comparison to the 0 h time point (21.85±2.47%) (p = 0.0006, n = 3). These cell death levels are maintained when analysed at 4 h post-OGD termination (50.55±3.14%, n = 3). In comparison, control cells are unaffected by equivalent treatments, in the presence of oxygen and glucose. (n = 3). Data represent mean ± SEM. (TIF) [file pone.0083717.s004.tif]

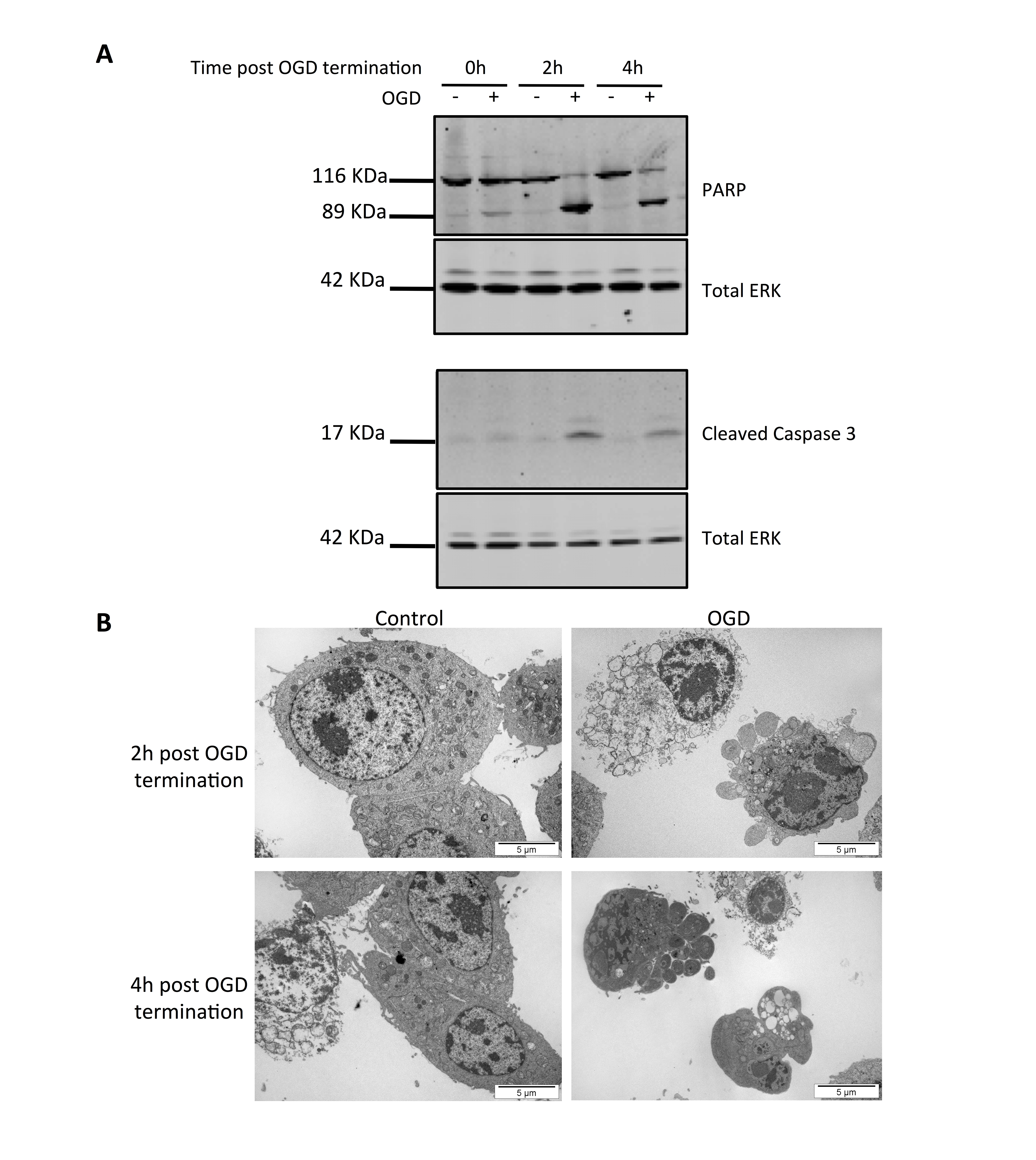

Supplement: Figure S5 — OGD in N2As induces cell death that displays characteristic hallmarks of apoptosis. A. N2As exposed to 4 h of OGD were examined for PARP cleavage and generation of the cleaved active caspase 3 fragment by Western analysis. The representative blots illustrate that OGD has induced a substantial increase in the cleaved PARP product of 89 kDa, characteristic of apoptosis, in comparison to the 0 h time point. Furthermore, this cleavage product is still present when examined at 4 h post OGD termination. Western analysis for the 17 kDa active cleavage product of caspase 3 also showed a large increase in its abundance at 2 h post-OGD termination, in comparison with the 0 h sample. The 17 kDA cleavage product declined in levels by 4 h post-OGD termination, when compared to the 2 h sample. The diagram represents a representative image from n = 3. B. N2As exposed to OGD show morphological characteristics of apoptosis. Electron microscopy was utilised to identify morphological hallmarks of apoptosis at 2 and 4 h post-OGD termination. Control images depict representative images of healthy cells. A small number of cells appear to be lysed (open arrow), illustrating basal death levels in the culture. At 2 h post-OGD termination cells with budding membranes (closed arrow) and condensed chromatin (dashed arrow) can be detected. Budding membranes and condensed chromatin are also detected at 4 h post OGD termination. (n = 1) Scale bar = 5 µm. (TIF) [file pone.0083717.s005.tif]
